# Authenticity and species identification of Fritillariae cirrhosae: a data fusion method combining electronic nose, electronic tongue, electronic eye and near infrared spectroscopy

**DOI:** 10.3389/fchem.2023.1179039

**Published:** 2023-04-28

**Authors:** Xin-Jing Gui, Han Li, Rui Ma, Liang-Yu Tian, Fu-Guo Hou, Hai-Yang Li, Xue-Hua Fan, Yan-Li Wang, Jing Yao, Jun-Han Shi, Lu Zhang, Xue-Lin Li, Rui-Xin Liu

**Affiliations:** ^1^ School of Pharmacy, Henan University of Chinese Medicine, Zhengzhou, China; ^2^ Department of Pharmacy, The First Affiliated Hospital of Henan University of Chinese Medicine, Zhengzhou, China; ^3^ Henan Province Engineering Research Center for Clinical Application, Evaluation and Transformation of Traditional Chinese Medicine, Zhengzhou, China; ^4^ Co-Construction Collaborative Innovation Center for Chinese Medicine and Respiratory Diseases by Henan and Education Ministry of China, Henan University of Chinese Medicine, Zhengzhou, China; ^5^ Henan Provincial Key Laboratory for Clinical Pharmacy of Traditional Chinese Medicine, Zhengzhou, China; ^6^ Zhengzhou Traditional Chinese Hospital of Orthopedics, Zhengzhou, China; ^7^ Engineering Research Center for Pharmaceutics of Chinese Materia Medica and New Drug Development, Ministry of Education, Beijing, China

**Keywords:** Fritillariae cirrhosae, data fusion, electronic nose, electronic eye, Electronic tongue, near infrared spectroscopy, authenticity, species

## Abstract

This paper focuses on determining the authenticity and identifying the species of *Fritillariae cirrhosae* using electronic nose, electronic tongue, and electronic eye sensors, near infrared and mid-level data fusion. 80 batches of *Fritillariae cirrhosae* and its counterfeits (including several batches of *Fritillaria unibracteata* Hsiao et K.C. Hsia, *Fritillaria przewalskii* Maxim, *Fritillaria delavayi* Franch and *Fritillaria ussuriensis* Maxim) were initially identified by Chinese medicine specialists and by criteria in the 2020 edition of *Chinese Pharmacopoeia*. After obtaining the information from several sensors we constructed single-source PLS-DA models for authenticity identification and single-source PCA-DA models for species identification. We selected variables of interest by VIP value and Wilk’s lambda value, and we subsequently constructed the three-source fusion model of intelligent senses and the four-source fusion model of intelligent senses and near-infrared spectroscopy. We then explained and analyzed the four-source fusion models based on the sensitive substances detected by key sensors. The accuracies of single-source authenticity PLS-DA identification models based on electronic nose, electronic eye, electronic tongue sensors and near-infrared were respectively 96.25%, 91.25%, 97.50% and 97.50%. The accuracies of single-source PCA-DA species identification models were respectively 85%, 71.25%, 97.50% and 97.50%. After three-source data fusion, the accuracy of the authenticity identification of the PLS-DA identification model was 97.50% and the accuracy of the species identification of the PCA-DA model was 95%. After four-source data fusion, the accuracy of the authenticity of the PLS-DA identification model was 98.75% and the accuracy of the species identification of the PCA-DA model was 97.50%. In terms of authenticity identification, four-source data fusion can improve the performance of the model, while for the identification of the species the four-source data fusion failed to optimize the performance of the model. We conclude that electronic nose, electronic tongue, electronic eye data and near-infrared spectroscopy combined with data fusion and chemometrics methods can identify the authenticity and determine the species of *Fritillariae cirrhosae*. Our model explanation and analysis can help other researchers identify key quality factors for sample identification. This study aims to provide a reference method for the quality evaluation of Chinese herbs.

## 1 Introduction

*Fritillariae cirrhosae* is an herb used both in traditional Chinese medicine and as food. *Fritillaria cirrhosae* is used in the treatment of cough, it eliminates phlegm, relieves asthma, reduces blood pressure, has analgesic effects, prevents ulcers, and has antibacterial and anti-inflammatory properties (Zhong et al., 2019; Chen et al., 2020). The sources of *Fritillariae cirrhosae* recorded in the 2020 edition of *Chinese Pharmacopoeia* include *Fritillariae cirrhosae*, *Fritillaria unibracteata*, *Fritillaria przewalskii*, *Fritillaria delavayi*, *Fritillaria taipaiensis*, *Fritillaria unibracteata*. These are named songbei, qingbei, lubei, *etc.*, based on different characteristics. The Fritillaria genus includes other related plants such as *Fritillaria thunbergii*, and *Fritillaria ussuriensis*. Because of the scarcity of *Fritillariae Cirrhosae* and the difficulties in cultivating it, it is common to find other plants sold as *Fritillaria cirrhosae* especially the cheap and easy to obtain *Fritillaria ussuriensis*. The presence of the market of plants sold as *Fritillariae Cirrhosae* would weaken the safety, efficacy and stability of clinical application of *Fritillariae Cirrhosae* decoctions. Therefore, efficient, rapid and sensitive authenticity and species identification technology is of great significance to ensure the quality of *Fritillariae Cirrhosae* decoction pieces (Xin et al., 2014; Hua et al., 2021).

Chinese herb medicines are traditionally identified by integrating a variety of human senses to determine their quality. This method is fast but subjective and difficult to quantify. The development of modern analytical techniques, chemical and biological detection techniques such as chromatography, spectroscopy, and molecular biology has played a key role in the identification and quality evaluation of TCM decoction pieces (Moon et al., 2018; Qi et al., 2018; Pu et al., 2019; Zhang Y. et al., 2021). Detection methods based on modern analytical techniques such as chromatographic methods have high accuracy, but the sample pretreatment is complex, time-consuming and costly. Artificial intelligence sensory technology can imitate human sensory systems, quantifying information and providing fast and accurate comprehensive information on the samples. Such methods are widely used in the detection and analysis of drugs and food (Buratti et al., 2018; Orlandi et al., 2019; Xu et al., 2019; Zhang X. et al., 2021).

Data fusion strategy consists in merging complementary information to obtain more data points; this strategy has been gradually applied to trace the origin of Chinese medicine (Shen et al., 2019; Wang et al., 2019; Jing et al., 2022), identify its quality (Ying et al., 2017; Dai et al., 2018; Sun et al., 2020) and analyze pharmaceutical processes (Wang et al., 2021; Zhang et al., 2022). Data fusion includes low, medium and high-level fusion. In low-level data fusion, the original data are directly combined into a new matrix. In mid-level data fusion, features are firstly extracted from the original data and then features are fused. It is worth noting that the removal of redundant information can improve the efficiency of the algorithm. In high-level data fusion, single data sources are firstly identified and chosen, and the final result is obtained based on the recognition results of each data source (Borràs et al., 2015). The flowchart of data fusion in this article is shown in Supplementary Data Sheet 1. The compositions of traditional Chinese medicine decoction pieces are complex, and the data measured by a single technology are not sufficient to accurately determine the authenticity and identify the species of the samples. Similar to what humans do, data fusion strategies can complement different sensory information to improve the identification accuracy. Previous studies have found that data fusion of observations made by artificial intelligence senses, such as electronic noses, electronic tongues and electronic eyes, can be successfully used to differentiate the two botanical origins of Magnolia Officinalis Cortex (Jing et al., 2022), evaluate the quality of Xiaochaihu granules (Zhang X. et al., 2021), identify products made with Curcuma (Lan et al., 2020), and identify and classify medicinal materials based on their smell and taste (Lan et al., 2020; Jing et al., 2022).

Principal Component Analysis-Discriminant Analysis (PCA-DA) and Partial Least Squares-Discriminant Analysis (PLS-DA) are two methods based respectively on principal component regression and partial least squares regression. The PCA-DA algorithm applies discriminant analysis (DA) based on principal component analysis (PCA), using the principle of principal component analysis to further compress high-dimensional data by maximizing the ratio of within-class variance and minimizing the ratio of between-class variance, thus exploring the combination of variables that can explain the main trends of the dataset (Chua et al., 2011; Wong et al., 2014). PCA-DA can simplify overlapping sample information in multi-dimensional data, and is more suitable for multi-class classification. PLS-DA can reduce the dimension of original data and simplify sample information. The mechanism of this technique is to search for linear combinations of the original variables (latent variables) that display maximum covariance with the Y-variables (classes) for classification prediction (Borraz-Martínez et al., 2019). A discriminator, or threshold is created to separate the different classes. The classification model is established by using known categories as a training set and then is used to predict the unknown samples. PLS-DA can determine whether the samples belong to a predefined category (Ballabio and Consonni, 2013; Yao et al., 2018; Borraz-Martínez et al., 2019). PLS-DA is generally used to deal with binary classification problems, but the PLS algorithm can deal with multi-column dependent variable Y, so PLS-DA can be used for multi-class classification in some cases.

In this study, we used a mid-level data fusion strategy to verify the authenticity and determine the species of *Fritillariae Cirrhosae*. Firstly, we analyzed the NIR spectra and the sample responses of electronic nose, electronic tongue and electronic eye. Based on four kinds of single source data (electronic nose, electronic eye electronic tongue and NIR), we then constructed PLS-DA models to determine the authenticity of the samples, and PCA-DA models to identify the species of the samples. Secondly, we selected the original variables (electronic sensors) respectively based on Variable Importance in Projection (VIP) and wilk‘s lambda value, and the NIR characteristic spectral bands based on Competitive Adaptive Reweighted Sampling (CARS). And the selected variables from three intelligent sensors were fused for three-source data fusion, the selected variables from four kinds of single source data were fused for four-source data fusion. Finally, based on the fusion variables data matrix, we verified the authenticity and identified the species of the samples. Also, we determined the optimal model based on the accuracies of models and analyzed it in combination with the sensor response signals. This study aims to provide a reference for the quality evaluation of *Fritillariae Cirrhosae* and other traditional Chinese medicine decoction pieces.

## 2 Materials and methods

### 2.1 Samples

**FIGURE 1:**
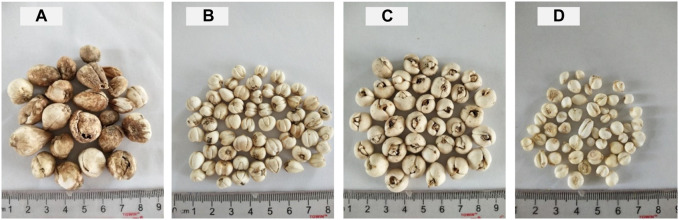
Samples plot: sample15, FU **(A)**; sample38, FP **(B)**; sample50, FD **(C)**; sample73, FUS **(D)**.

80 batches (20 of *Fritillaria unibracteata* (FU), 20 of *Fritillaria przewalskii* (FP), 20 of *Fritillaria delavayi* (FD), and 20 of *Fritillaria ussuriensis* (FUS)) were collected from either the Zhengzhou traditional Chinese medicine hospital, the Zhengzhou Chinese medicine market, the first affiliated hospital of Henan University of Chinese medicine or the Bozhou Chinese medicine market. Each batch consisted of 100 g of material. Samples plot is shown in Figure 1.

### 2.2 Sample identification

#### 2.2.1 Human experience: Specialist identification

Experts with a wealth of experience in identifying TCM decoction pieces (over 15 years work experience in the production, processing and preparation of TCM decoctions), affiliated with different organizations (universities, industries, hospitals, etc.), and with different backgrounds (covering the cultivation, processing, circulation and use of TCM decoction pieces), were invited to identify the samples. A total of 8 experts from the Henan province identified the samples.

#### 2.2.2 Physicochemical identification based on pharmacopoeia

The samples were identified based on their appearance, by microscopic identification, thin-layer chromatography (TLC), moisture content, ash content and other detection methods as described in the *Fritillariae Cirrhosae* section in the 2020 edition of Chinese Pharmacopoeia.

### 2.3 Electronic sensory signal acquisition and preprocessing

#### 2.3.1 Electronic nose

Olfactory information collection was acquired by ten types of metal oxide sensors (W1C, W5S, W3C, W6S, W5C, W1S, W1W, W2S, W2W, and W3S) from German PEN3 electronic nose (PEN3 portable electronic nose by the German AIRSENSE company). 2 g powder were taken from samples 1 to 80 and 3 replicates per sample, marked as A-1, A-2, A-3. Based on our pre-experimental results, the samples were tested after 15 min. The experiment was carried out at 20°C and 60% humidity. The sampling conditions were the following: sampling time (the time the sample was exposed to the sensors), 120 s; cleaning time, 100 s; sampling interval, 1 s; air intaking flow rate, 150 mL·s^-1^. An olfactory information matrix *X*1 (80 × 10) was obtained, and the data was used to establish the model.

#### 2.3.2 Electronic eye

The IRIS VA400 electronic eye (France Alpha MOS company) was used to collect visual information on the samples. An area of about 8 × 8 cm^2^ was randomly selected from each sample and placed on watch glass. Top lighting conditions were selected based on pre-experimental results, and a 24-color color correction plate was used for color correction. A 5 mm aperture was used and the upper and lower backlights were simultaneously turned on to eliminate the background. Images from each sample were collected three times after changing the position of the samples. A visual information matrix *X*2 (80 × 65) was obtained by 65 sensors, and the data was used to establish the model.

#### 2.3.3 Electronic tongue

Taste information was collected using the TS-5000Z Insent electronic tongue (Ensoul Technology LTD.). The C00, AN0, BT0 and AE1 sensors were used. The principle of the electronic nose is to use sample gas to interact with the sensor to change the conductivity of the active material of the sensor, thus generating the response value. 5 g of each sample were weighed and crushed in an electric homogenizer for 15 s. The sample powder was then placed in 100 mL of artificial saliva to be ultrasonically processed. The samples were subsequently filtered, sterilized and poured into a special cup to be tested by the electronic tongue. The electronic tongue sensor was cleaned in a cleaning solution for 90 s, in a reference solution for 120 s, and in a different reference solution for 120 s. The sensor started to collect sample information after the response value stabilized at 0 for 30s. The acquisition time of the beforetaste value of each sample was 30 s, the sensors were then cleaned for 3 s in the two reference solutions. Finally, the sensors were inserted into the new reference solution to collect data for 30 s and the aftertaste value was exported. This cycle was repeated four times, data from the first cycle was removed, and the average data of the last three cycles was calculated. Liquid used for cleaning, balancing and aftertaste-testing were placed in different sample cups. A six sensor taste information matrix *X*3 (80 × 6) was obtained and the original data were used to establish the model.

#### 2.3.4 NIR spectra acquisition and spectra selection

(1) NIR spectra acquisition: NIR spectra were acquired by the Nicolet6700 Fourier transform near-infrared spectrometer (InGaAs detector). Sampling mode was set to diffuse reflection. The samples were dried in an oven at 60°C for 6 h and then crushed and sieved with the No.4 Pharmacopoeia sieve (250 ± 9.9 μm). The parameters of the NIR spectrometer were the following: reference: air; temperature: 25°C ± 2°C; relative humidity: 50 %–60%; resolution: 8cm^-1^; number of scans: 64; scanning range: 12,000 cm^-1^-4,000 cm^-1^; number of gratings: 9–11. Each sample was placed in a quartz sample pool and scanned three times. The spectrum information was collected at room temperature by the opus spectrum acquisition software (Bruker company) and the average spectra were calculated. A NIR spectral information matrix *X*4 (80 × 2075) was obtained.
(2) Spectra selection: The Competitive Adaptive Reweighted Sampling (CARS) method was used to eliminate redundant information in the NIR spectra and to select the characteristic spectra related to the structure of the tested compounds. CARS uses Monte Carlo sampling to establish a Partial Least Squares (PLS) model and simulates the principle of survival of the fittest to eliminate variables by exponential decay function, so that the wavelength variables with smaller absolute values of regression coefficients in the PLS model are removed and the wavelength points with larger weights are screened out through adaptive reweighted sampling technology. An optimal variable wavelength subset was selected based on Root Mean Squares Error of Cross-Validation (RMSECV) of the PLS model (Li et al., 2009; Wang et al., 2017). When using CARS, the number of iterations of Monte Carlo was set to 100, and the pretreatment method of NIR spectra data was “mean centering”.

### 2.4 Construction of authenticity and species identification model based on single source data

PLS-DA was used to establish the authenticity identification models of *Fritillariae cirrhosae* based on data from electronic nose, electronic tongue, electronic eye, and NIR spectra. The performance of the four models based on each type of sensor was evaluated with leave-one-out cross-validation. Because PLS-DA has unclassified cases in multi-classification (Rui-xin et al., 2020; Wen-hao and Shi, 2021), PCA-DA was then chosen to establish the species identification models of *Fritillariae cirrhosae* based on electronic nose, electronic tongue, electronic eye and NIR spectra. The performance of the models was evaluated by the model’s accuracy (the ratio of the number of correctly classified samples to the total number of samples) after leave-one-out cross-validation.

### 2.5 Variable selection

Variable Importance in Projection (VIP) of each sensor in the authenticity identification model was obtained with the PLS-DA algorithm. VIP can explain the contribution extent of independent variables to dependent variables. The larger the VIP is, the greater the contribution of independent variables compared to dependent variables is. VIP >1 indicates a significant contribution of independent variables to dependent variables. In the identification of authenticity, the original variables with VIP greater than 1 were selected from electronic nose, electronic tongue and electronic eye. Wilk’s lambda value represented the ratio of within-group variation to between-group variation in the training set (Huan-ran et al., 2019). The smaller one variable’s wilk’s lambda value is, the stronger the discriminant ability of this variable is. In species identification, by gradually eliminating the variables with the largest Wilk‘s lambda value in the PCA-DA model, we selected characteristic variables from electronic nose, electronic tongue and electronic eye sensors according to the change of model’s accuracy after removal of different variables. Key wavelengths selected by CARS were used as NIR characteristic variables.

### 2.6 Construction of authenticity and species identification model based on fusion data

Based on the sample identification results, we constructed an authenticity PLS-DA model and a species PCA-DA model using the fusion of three-source intelligent sensors and three-source intelligent sensors and NIR (based on the variables selected in 2.5). The performance of the model was evaluated considering the accuracy of the model after leave-one-out cross validation.

### 2.7 Model explanation and analysis

Based on the VIP and Wilk’s lambda value of the optimal discriminant model, we identified the sensors that most contributed to the classification. We analyzed the characteristic component and key quality factors affecting the authenticity and species identification of *Fritillariae cirrhosae*.

## 3. Results and discussion

### 3.1 Sample identification

The results of the specialist identification are shown in Supplementary Table S1. When the identification results of 8 specialists are inconsistent, we determined the final specialist identification result of each sample by judging that the number of specialists was whether larger than or equal to 3/4 of the total specialists or not.

The identification results of *Fritillariae cirrhosae* based on the 2020 edition of Chinese Pharmacopoeia were the following.

• Appearance characteristics: S23, S24, S43 and S61-S80 did not meet the requirements;
• Microscopic identification: S71, S74 and S78 did not contain spiral vessel, while all other samples met the requirements of the pharmacopeia to be identified as F. cirrhosae;
• Thin-Layer Chromatography identification: S23 and S24 did not contain peiminine and therefore were not identified as F. cirrhosae, while TLC of S61-S80 showed the same color spots corresponding to the reference medicinal materials of *Fritillariae cirrhosae*;
• The results of moisture and ash content for all 80 samples were in line with the requirements of the pharmacopeia for the identification of F. cirrhosae.

After combining the identification results of the specialist identification and the test results based on the 2020 edition of the Chinese Pharmacopoeia, (if the results of artificial experience identification and the pharmacopeia test were in disagreement, we carried out a retest to avoid identification errors), the final results of our identification were: S1-S20, FU; S21-S22, S25-S40, FP; S41-S42, S44-S60, FD; S23, S24, S43, S61-S80, FUS.

### 3.2 Signal response of electronic senses

**FIGURE 2:**
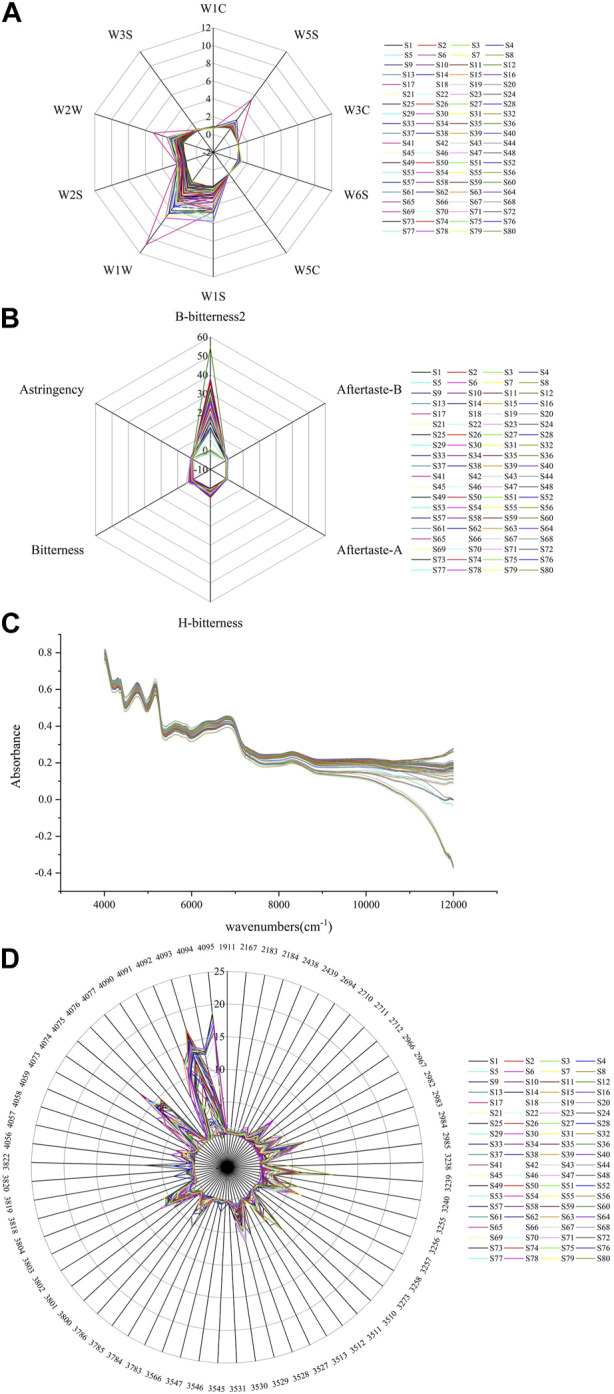
response values **(A)** electronic nose, **(B)** electronic tongue, **(C)** NIR spectra, **(D)** electronic eye.

Based on the response of the electronic sensors, it can be seen that in the electronic nose test (Figure 2A) most samples had the largest response value on W1W sensor, followed by W1S and W2W, while sample S69 had the largest response value for the W1W, W2W and W5S sensors. All samples had small response values on the W5C, W3C and W1C sensors, which are sensitive to aromatic compounds. In the electronic tongue test (Figure 2B), samples had the largest response value at the B-bitterness2 sensor, which is related to the alkaloid components contained in the samples. The NIR spectra (Figure 2C) showed that the samples had more abundant information at wavelengths 4,000–7,000 cm^-1^. In the electronic eye test (Figure 2D), the samples had the largest response at color number values of 4,095, 4,093, 4,094, 4,092 and 4,075, which are related to white surfaces characteristics of *Fritillariae cirrhosae* and *Fritillaria taipaiensis*.

### 3.3 Selection of NIR spectra

The full NIR spectra data for the 80 samples were selected by CARS. After 81 iterations, the Root Mean Square Error of Cross-Validation (RMSECV) of the PLS model was the smallest. Eight key wavelengths were eventually selected. The number of wavelengths decreased significantly from 2075 to 8. The eight key wavelengths of the NIR spectra were 4,188 cm^-1^, 5,102 cm^-1^, 5,970 cm^-1^, 6,900 cm^-1^, 9,754 cm^-1^, 10,884 cm^-1^, 11,254 cm^-1^, and 11,678 cm^-1^.

**FIGURE 3:**
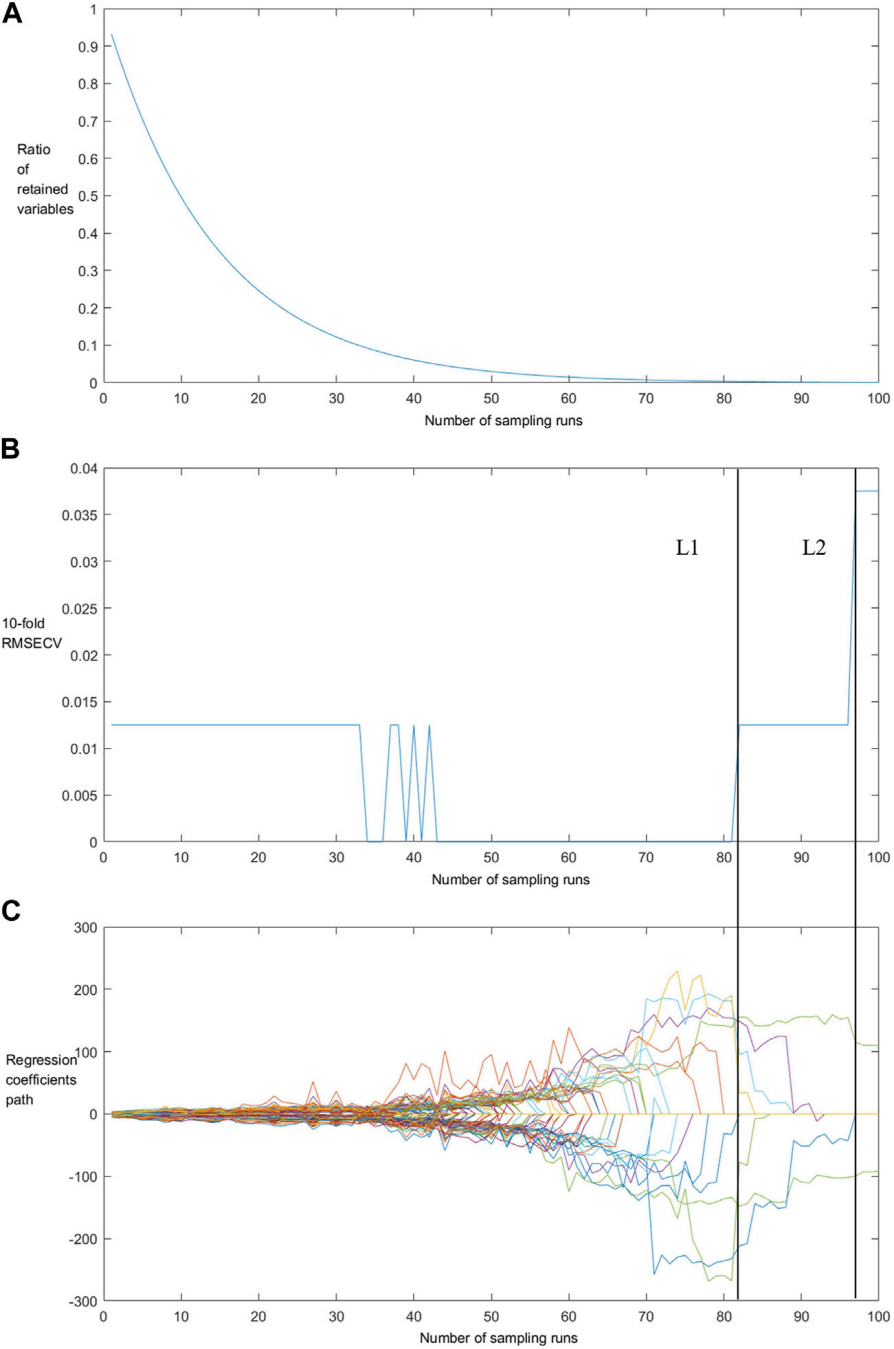
The changing trend of the ratio of retained variables **(A)**, 10-fold RMSECV values **(B)** and regression coefficients of each variable **(C)** with the increasing of sampling runs.

As shown in Figure 3A, there were two operation stages of CARS: fast selection (sampling times 0–30) and refined selection (sampling times 30–80). In the fast selection stage, the exponentially decreasing function filters out the wavenumbers with little or no information, thus effectively simplifying the spectral data (Li et al., 2009; Ma Hui et al., 2021).

As shown in Figure 3B, the RMSECV remains unchanged in the rapid selection stage, while in the initial stage of refined selection it changes from the maximum to the minimum due to the removal of key variables during the iteration process. As shown in L1 and L2 of Figure 3, when the variables marked L1 were filtered and removed, the regression coefficient of one variable was also immediately reduced to 0 under this sampling number, indicating CARS removed variables that played a key role in the PLS model as part of the sampling operation, so that there was a sharp decline in model’s stability. This phenomenon can also be seen in L2. Therefore, the variables selected by CARS are called “key variables”.

### 3.4 Authenticity identification

#### 3.4.1 Electronic nose

**FIGURE 4:**
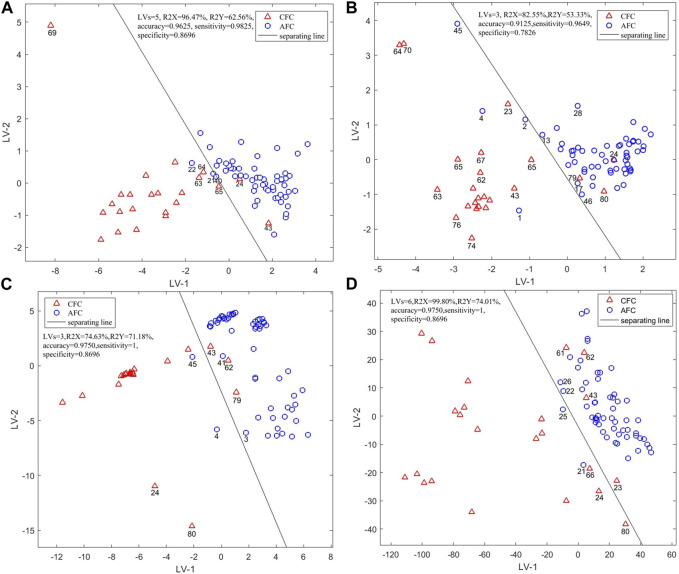
Scores Plot of Single-Source PLS-DA authenticity and counterfeit identification model based on **(A)** electronic nose, **(B)** electronic tongue, **(C)** electronic eye and **(D)** NIR spectra.

PLS-DA was used to establish a qualitative identification model to identify 60 batches of *Fritillariae cirrhosae* and 20 batches of *Fritillaria taipaiensis* based on data collected by electronic nose. Five latent variables, which explain 96.4% of the total variation in the *X* data among the samples, can be used to establish a PLS-DA model. As shown in Figure 4A, the two types of samples displayed obvious cluster characteristics, while sample S69 was significantly far from all other counterfeit samples. The electronic nose test was then repeated and its results showed a considerable difference in three-times sensory data for S69, this may have been the result of differences in the decoction due to its complex sources.

Near the separation line, under the first two latent variables, S24 and S43 were misclassified as authentic FC, this can be explained from the results of previous human experience of specialist identification and the pharmacopeia detection: in the identification and detection results of S24, four experts had identified it as counterfeit, one expert identified it as authentic, and three other experts failed to identify it. And the results of TLC and appearance test from the pharmacopeia both showed that S24 could not be identified.

In the identification and detection results of S43, three experts had identified it as authentic and five experts had failed to identify it. The pharmacopeia detection results showed that S43 was counterfeit. S21, S22 and S40 of FP were identified as counterfeits, so it was speculated that the misclassified samples were caused by differences in geographical sources. The accuracy of this model was 96.25%, and the sensitivity (*Se*), specificity (*Sp*) after cross-validation were 0.9825 and 0.8696. These parameters indicate that the electronic nose can identify both FC and FUS.

#### 3.4.2 Electronic tongue

The first three latent variables were used to establish a better PLS-DA qualitative analysis model, which could explain 82.55% of the total variation in the samples. The distribution of counterfeit samples was relatively scattered, indicating that the counterfeits have larger internal differences in terms of intelligent taste information. As shown in Figure 4B, using the first two latent variables S24 was misclassified as again, and so were S79 and S80, while S1, S2, S4 and S45 were misclassified as counterfeits. In the specialist identification of S79, only three experts had identified it as FUS, suggesting it was a difficult sample to identify. S1, S2 and S4 were in line with regulations in the pharmacopeia of FC and the detection indexes of S80 were in accordance with the standards of FUS, while these samples were still mis-classified under the first two latent variables. We speculated that the internal material of the samples had changed because of variations in the environmental temperature and humidity during storage and transportation. The appearance detection of S45 from the pharmacopeia identification did not meet the requirements, and the TLC results were not obviously colored. We speculated that these differences caused its misclassification. The accuracy of the PLS-DA model established by the electronic tongue sensors was 91.25%, and *Se* and *Sp* after cross-validation were 0.9649 and 0.7826. These parameters indicate that the model performance needs to improve to be able to use electronic tongue sensors to identify FC and FUS.

#### 3.4.3 Electronic eye

After leave-one-out cross-validation, the qualitative analysis model established by the first three latent variables had the best performance. The first three latent variables could explain 74.63% of the total sample variation. From Figure 4C, the cluster characteristics of authentic and counterfeit samples are not obvious. Samples S4, S45, S43 and S79 were misclassified as in the previous tests, indicating that the response value of these samples was significantly different from other samples. Although there were some mis-classified samples using the first two latent variables, when the number of latent variables in the model is three, the accuracy of the model reaches 97.50%, and the model parameters *Se* and *Sp* are 1,0.8696 and 0.9750. These parameters indicate that the electronic eye can identify the two types of samples.

#### 3.4.4 NIR spectra

When the first six latent variables were selected for modeling, the model performance was the best. The first six latent variables can explain 99.80% of the total variation among the samples. From Figure 4D it can be seen that the cluster characteristics of counterfeit samples were not obvious. In addition to the samples that have been misclassified, S23, S25 and S61 were also misclassified under the first two latent variables.

Two experts had identified S23 as counterfeit, one expert had identified it as authentic and five experts had failed to identify it, while the results of the extract test from the pharmacopeia and appearance detection showed that S23 was unqualified. The pharmacopeia detection indexes of S25 and S61 were in line with the regulations respectively of FC and FUS. The overall classification results of the model were determined by the six latent variables. The accuracy of this model was 97.50%. *Se* and *Sp* were 1 and 0.8696. These parameters indicate that the full spectra, combined with the PLS-DA algorithm, can accurately identify the samples.

#### 3.4.5 Variable selection of authenticity identification model

Original variables with VIP >1 in the four single-source PLS-DA models are shown in Table 1.

**TABLE 1:** Original variables with VIP >1.

Data Source	Original Variables
electronic nose	W1C, W3C, W5C, W1S, W2S
electronic tongue	Aftertaste-A, H-bitterness,Bitterness, Astringency
electronic eye	Color number value 2,712、 2,985、3,257、3,258、3,273、3,513、3,529、3,530、3,545、3,783、3,786、3,800、3,802、3,803、3,818、4,056、4,057、4,059、4,073、4,074、4,076、4,090、4,091、4,094、4,095
NIR	Wavenumber 4188.696、5102.804、5970.628、6900.164、9754.34、10884.44、11254.71、11678.98

#### 3.4.6 Three-source data fusion

**FIGURE 5:**
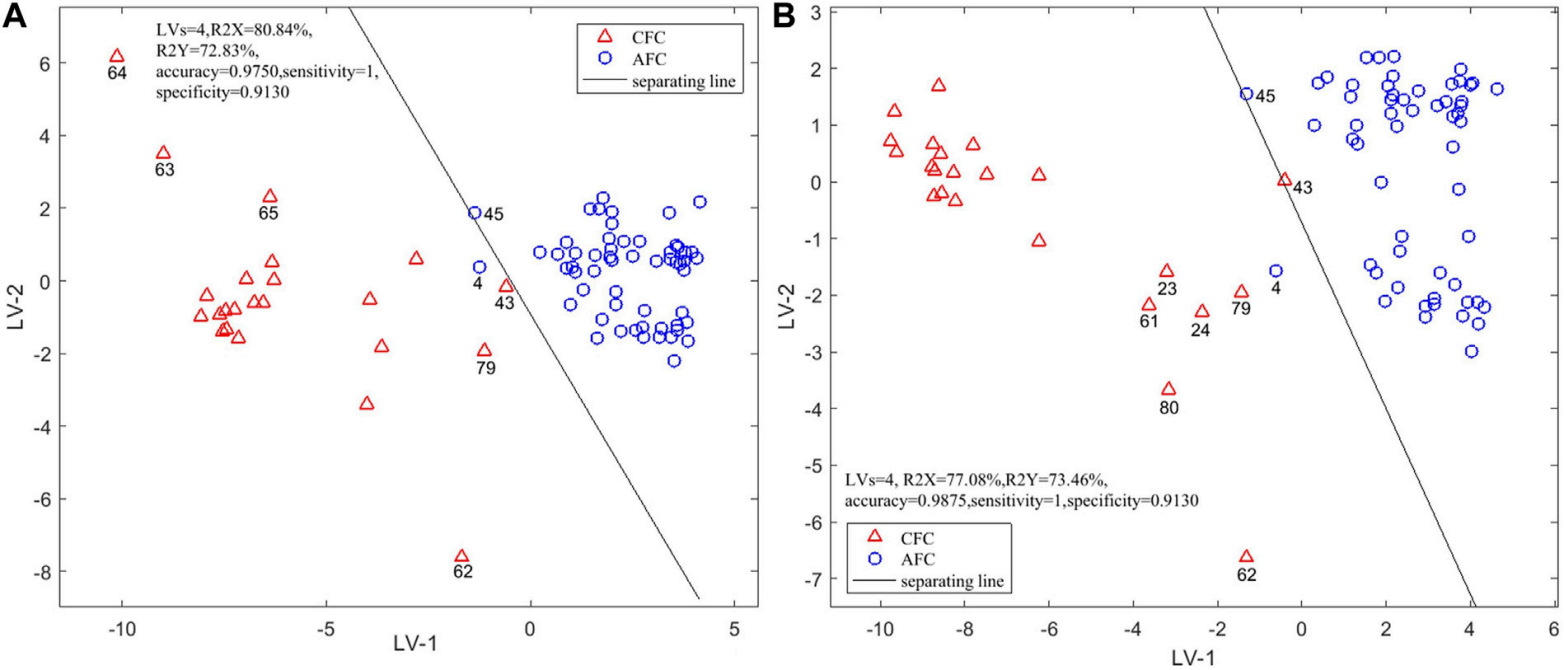
Scores Plot of PLS-DA authenticity and counterfeit identification model based on three-source fusion **(A)** (EN + ET + EE) and four-source fusion **(B)** (EN + ET + EE + NIR).

The original variables (sensors) with VIP >1 in each single-source intelligent sensory data model were fused to explore and analyze the discriminant ability of the fused data in identifying the samples. When the first four latent variables were selected for modeling, the model performance was the best: the first four latent variables explain 80.84% of the total variation among the samples. As in Figure 5A, the cluster characteristics of two types are more obvious than those in single-source data analysis, and only S4 was misclassified as FUS on the first two latent variables. The rate of correct results of the model after three-source fusion was 97.50%. Although the accuracy is the same as using the electronic eye sensor or NIR alone, the accuracy is greater than using the electronic tongue or the electronic nose alone. The classification results on the first two latent variables were also better than that when using single-source electronic sensory data alone. The model parameters, *Se* and *Sp*, were 1 and 0.9130.

These results show that data fusion can obtain multi-dimensional information of samples, and the fusion of original variables will contribute to sample classification.

#### 3.4.7 Four-source data fusion

The sample information obtained by the fusion data is richer, and the model’s classification performance is improved. We therefore explored the result of the fusion of NIR feature spectra and data from three electronic sensors. After leave-one-out cross-validation, the model established by the first four latent variables had the best performance. These four latent variables explained 77.08% of the variation among samples. In Figure 5B it can be seen that the classification situation after four-source data fusion is similar to that after three-source data fusion. Although S4 and S43 were misclassified under the first two latent variables, the model performance was determined by the first four latent variables and the accuracy of the model constructed with the first four latent variables was 98.75%, which is higher than when using any single source. The model parameters *Se* and *Sp* were 1 and 0.913. The PLS-DA model constructed with four-source data fusion has therefore the best performance and achieved the best classification.

**FIGURE 6:**
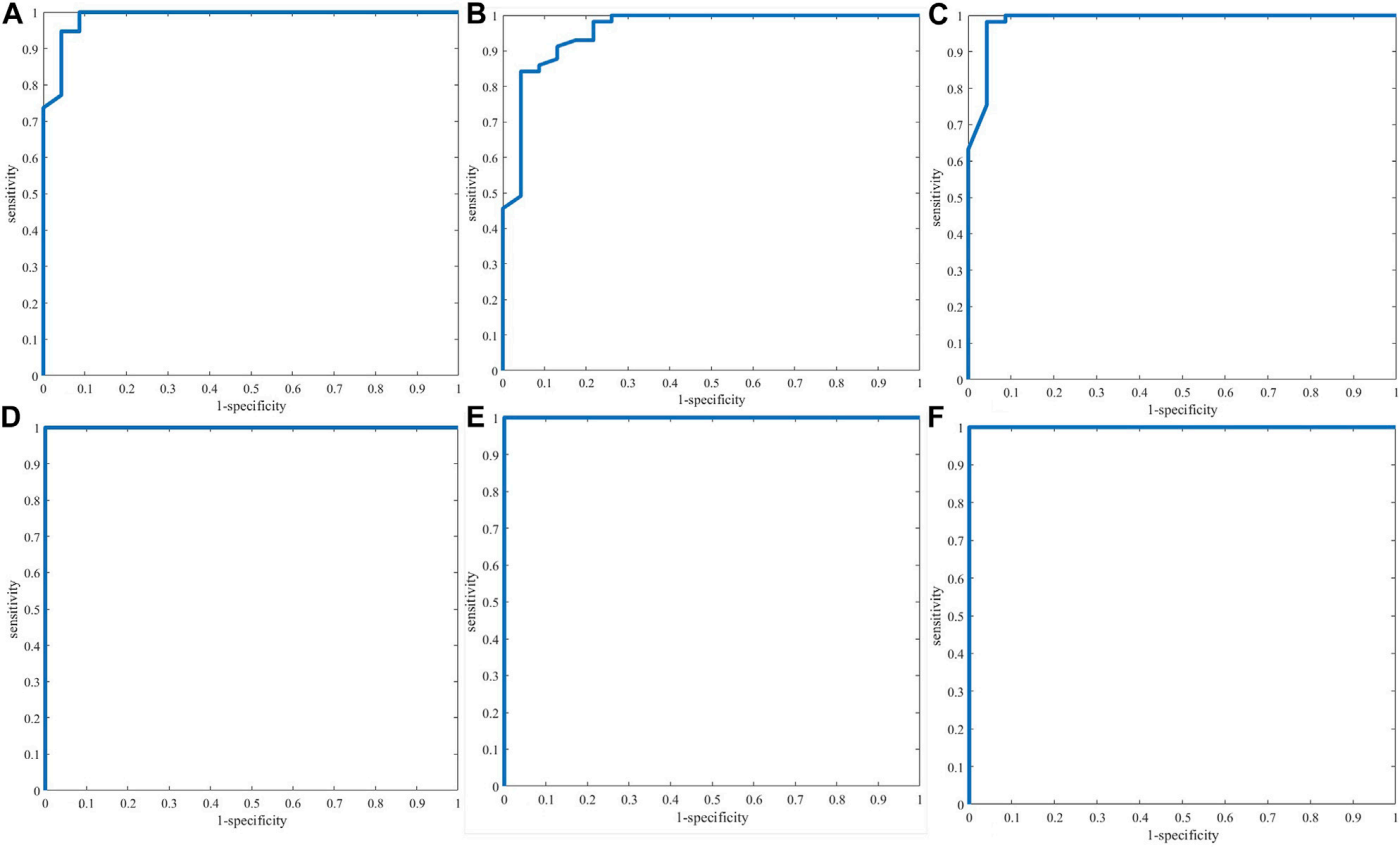
ROC curves of single-source PLS-DA authenticity and counterfeit identification model based on electronic nose **(A)**, electronic tongue **(B)**, electronic eye **(C)** and NIR spectra **(D)** and three-source fusion **(E)** (EN + ET + EE) and four-source fusion **(F)** (EN + ET + EE + NIR).

ROC curves (Figure 6) showed that PLS-DA authenticity and counterfeit identification models based on NIR and three-source data fusion and four-source data fusion have better performance and the AUC of the three models is 1 while the classification performance of PLS-DA model based on electronic tongue (AUC:0.9573) is not very well in comparison with the other five models.

### 3.5 Species identification

#### 3.5.1 Electronic nose

**FIGURE 7:**
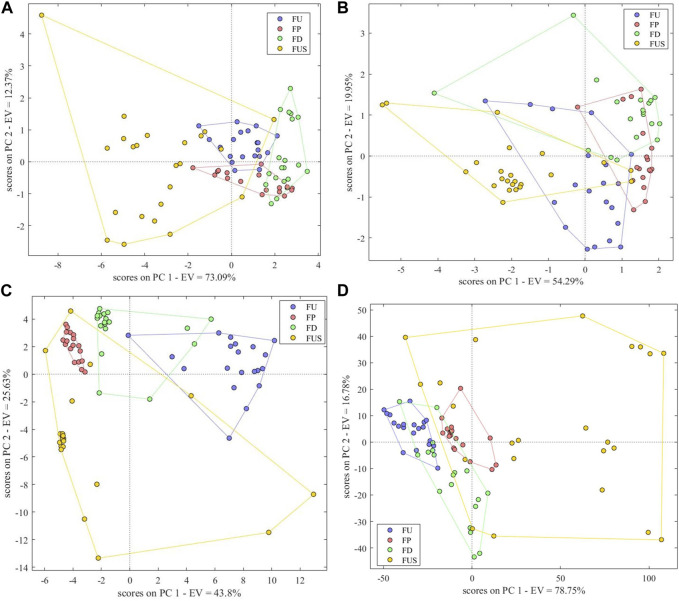
Scores Plot of Single-Source PCA-DA species identification model based on **(A)** electronic nose, **(B)** electronic tongue, **(C)** electronic eye and **(D)** NIR spectra.

The accuracy of the PCA-DA model based on electronic nose data was 85%, and there were no unclassified samples. The model parameters *Se*, *Sp* and *Pre* were respectively 0.85, 0.98 and 0.94. The classification results showed that 3 FU were misclassified as FD, 3 FP were misclassified as FD, 1 FP was misclassified as FUS, 1 FD was misclassified as FP, 1 FUS was misclassified as FU, 2 FUS were misclassified as FP and 1 FUS was misclassified as FD. There was no obvious difference between different FC and FUS samples based on electronic nose data, resulting in a number of misclassified samples (Figure 7A). As shown in the scores plot, on the variation information represented by the first two principal components, FU and FD samples could be distinguished easily and the clusters of FU, FP and FD samples were clear, while counterfeit samples had a wide range of differences and appeared scattered.

#### 3.5.2 Electronic tongue

The correct rate of the PCA-DA model based on electronic tongue data was 71.25%, with no unclassified samples. The model parameters *Se*, *Sp* and *Pre* were respectively 0.65, 0.92 and 0.72. There were 7 FU samples misclassified, 6 FP, 5FD, and 5 FUS, indicating that electronic tongue data showed little differences between different FC and FUS samples. It can be also seen that the cluster distribution of the four types of samples was not obvious in the variation information represented by the first two principal components, and there were overlaps between the four types (Figure 7B). We conclude that the performance of the species identification model based on electronic tongue data is not good.

#### 3.5.3 Electronic eye

The PCA-DA model based on electronic eye data could effectively distinguish the four species of samples, and the correct identification rate we observed was 97.5%. The model parameters *Se*, *Sp*, and *Pre* were respectively 1.00, 0.98, and 0.95. All FU and FP samples were correctly classified, and there was only one misclassified sample in FD and one in FUS. As shown in Figure 7C, the cluster of FP samples was the clearest, followed by the FD and FU samples. On the contrary, there were some scattered samples in FUS, indicating that the traits of these samples were significantly different from other samples.

#### 3.5.4 NIR spectra

The PCA-DA model based on NIR spectra could also distinguish between the four types of samples. The correct identification rate was 97.5%, and parameters *Se*, *Sp*, and *Pre* were respectively 1.00, 0.98, and 0.95. FU, FP, and FD samples were all correctly classified. One FUS sample was misclassified as FU and another was misclassified as FD. The scores plot (Figure 7D) shows clearer clusters for FU and FP samples, while FD and FUS samples appear more dispersed in comparison.

#### 3.5.5 Variable selection of the species identification model

The variables from single-source data were selected based on the Wilk’s lambda value of the model. We proceeded by gradually removing variables with larger Wilk’s lambda values and stopped the removal at the point at which removing a variable resulted in the correct identification rate decreasing. The variables remaining were selected. Table 2 shows the variables selected for each intelligent sensor.

**TABLE 2:** Selected variables for electronic nose, electronic tongue and electronic eye.

Data Source	Original Variables
electronic nose	W1C, W3C, W6S, W5C, W1S, W1W, W2S, W2W, W3S
electronic tongue	H-bitterness,Bitterness, Astringency
electronic eye	Color number value 1911,2167,2183,2184,2438,2439,2694,2710,2711,2712,2966,2967,2982,2983,2984,2985,3239,3240,3255,3256,3257,3258,3512,3513,3529,3530,3531,3785,3786,3800,3802,3803,3818,4073,4074,4075,4076,4090,4091,4092,4093,4094,4095

#### 3.5.6 Three-source data fusion

**FIGURE 8:**
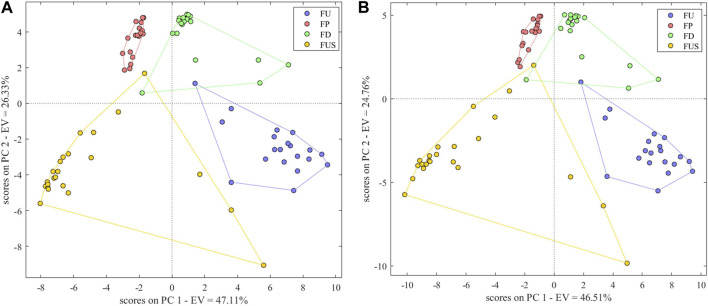
Scores Plot of PCA-DA species identification model based on three-source fusion **(A)** (EN + ET + EE) and four-source fusion **(B)** (EN + ET + EE + NIR).

Fusing data obtained from electronic nose, electronic tongue and electronic eye, the correct identification rate of the model was 95%, higher than that of the models using either only electronic nose data or only electronic tongue data. The model parameters *Se*, *Sp* and *Pre* were respectively 0.95, 0.98, and 0.90. The principal component score plot (Figure 8A) shows the cluster of FP samples is more concentrated than other samples. FU, FD and FUS clusters could be better together except for several samples. S2 was misclassified as FD; S45 was misclassified as FP; S43 was misclassified as FP and S79 was misclassified as FU. These samples were also misclassified in the authenticity and counterfeit identification. All other samples were correctly identified. The positive identification rate of the model based on fusion data was lower than that of the model based on electronic eye data alone, which we hypothesized could be explained by the removal of key variables during the process of variable selection. This suggests that the increase in the number of variables in data fusion does not necessarily improve the performance of the model, while it is crucial to focus on the choice of the variables.

#### 3.5.7 Four-source data fusion

After fusing the data of electronic nose, electronic tongue, electronic eye and near infrared, the correct identification rate of the model was 97.50%, and the model parameters *Se*, *Sp* and *Pre* were 1.00, 1.00, and 0.95. The principal component scores plot (Figure 8B) was similar to the scores plot of the three-source fusion data model. S43 was misclassified as FP and S79 was misclassified as FU, suggesting that the fusion of NIR spectra features and intelligent senses didn’t significantly improve the model performance and it can only reach the same classification ability of electronic eye and NIR.

Comparing the model parameters of single-source data and multi-source data model (Table 3), we observed that the parameters of the model after data fusion were equal to or better than in the single-source data models. In the authenticity and counterfeit identification model, the positive identification rate in the four-source fusion model was higher than that of each single-source data model. While in the species identification model, the parameters of the fusion model were only better than the single-source models based on data from either electronic nose or electronic tongue, but equal to the single-source model constructed from electronic eye sensors and NIR spectra. We believe this may be due to the fact that the accuracy of the identification model based on these two instruments was already high.

**TABLE 3:** Model parameters of single-source data and multi-source data (EN: electronic nose; ET: electronic tongue; EE: electronic eye) (the bold values means the parameters of optimal PCA-DA and PLS-DA models).

Model		parameters		
		Sp	Se	Ac
PLS-DA authenticity and counterfeit identification	EN	0.8696	0.9825	0.9625
	ET	0.7826	0.9649	0.9125
	EE	0.8696	1.0000	0.9750
	NIR	0.8696	1.0000	0.9750
	EN + ET + EE	0.9130	1.0000	0.9750
	**EN + ET + EE + NIR**	**0.9130**	**1.0000**	**0.9875**
PCA-DA species identification	EN	0.9800	0.8500	0.8500
	ET	0.9200	0.6500	0.7125
	**EE**	**0.9800**	**1.0000**	**0.9750**
	**NIR**	**0.9800**	**1.0000**	**0.9750**
	EN + ET + EE	0.9800	0.9500	0.9500
	**EN + ET + EE + NIR**	**1.0000**	**1.0000**	**0.9750**

### 3.6 Explanation and analysis of the models

#### 3.6.1 Explanation and analysis of the models based on VIP

**FIGURE 9:**
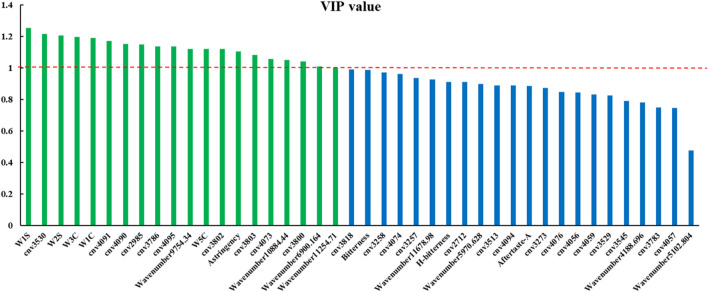
VIP value of each variable in the four-source fusion PLS-DA model (cnv: color number value).

We identified the optimal discriminant model as the model based on four-source data fusion, based on its accuracy. VIPs of each variable in this model are displayed in Figure 9. There were 20 variables with VIP >1, contributing to the classification of samples. Among these, the five sensors that played a key role in the classification based on electronic nose single-source data contributed greatly to the classification of authentic and counterfeit samples after data fusion. Among the eight NIR wavelengths selected by CARS, four of them had a greater contribution to sample classification after data fusion. There were 25 sensors that played an important role in the classification of electronic eye single-source data, while only 10 played a crucial role in authenticity and counterfeit classification after data fusion. Among the four sensors which contributed to the classification based on electronic tongue, only the “Astringency” sensor had VIP>1. We therefore concluded that the four instruments played different and complementary roles in the classification of sample authenticity and counterfeit.

The sensor with highest response in the electronic tongue was B-bitterness2, but its contribution to sample classification was relatively small. Although the sensor Astringency had a small response, it contributed greatly to sample classification.

We performed a *T*-test on the response values of the Astringency sensor for authentic and counterfeit samples and found there was a significant difference between the two types of samples (*p* < 0.05).

Among the sensors of the electronic nose, W1S, W2S, W3C, W1C and W5C contributed the most to the classification of authentic and counterfeit samples. The response values of all other sensors except W1S were small. In addition, sensors with larger response values (W1W, W2W and W5S) showed little contribution to the classification of samples. The response values of authentic and counterfeit samples were significantly different (*p* < 0.05) on W1S, W2S, W3C, W1C and W5C sensors.

Among the ten color number values that played an important role in the classification based on electronic eye data, the response values of the samples were all small for all values except 4,095, 3,802 and 3,803. The response values of the two types of samples were significantly different (*p* < 0.05). We found that the sensors that contributed the most to the sample classification were not necessarily the sensors with the highest response values. Some components had a smaller response value on the sensors, but, as the content of these components was significantly different in authentic and counterfeit samples, they played a key role in classification of the samples. This kind of component can be defined as “intelligent sensory information with high identification contribution”.

The response substances of sensors with VIP >1 are shown in Table 4, which indicates that their response values had a great influence on the classification of authentic and counterfeit FC. The response substances of these sensors can be used as key index components to determine the authenticity of FC.

**TABLE 4:** Response substances (information) of variables with VIP >1.

Variables	Response substances (information)
W1S	alkane
Color number value 3,530	L: 81.324, a:2.059, b:18.207
W2S	Alcohols, part of aromatic compounds
W3C	Ammonia, aromatic molecules
W1C	aromatic hydrocarbons
Color number value 4,091	L: 96.445, a: 7.26, b:30.891
Color number value 4,090	L: 96.225, a: 8.707, b:38.525
Color number value 2,985	L: 69.923, a: 4.012, b:10.507
Color number value 3,786	L: 82.772, a: 8.202, b:20.463
Color number value 4,095	L: 97.579, a: 0, b:0
W5C	alkene, aromatic compounds, polar molecules
Color number value 3,802	L: 86.778, a: 0.367, b:25.861
Astringency	astringency
Color number value 3,803	L: 87.04, a: 1.983, b:17.941
Color number value 4,073	L: 91.97 a: 2.515, b:40.97
Color number value 3,800	L: 86.343 a: 2.375, b:41.459

#### 3.6.2 Explanation and analysis of the models based on Wilk’s lambda

**FIGURE 10:**
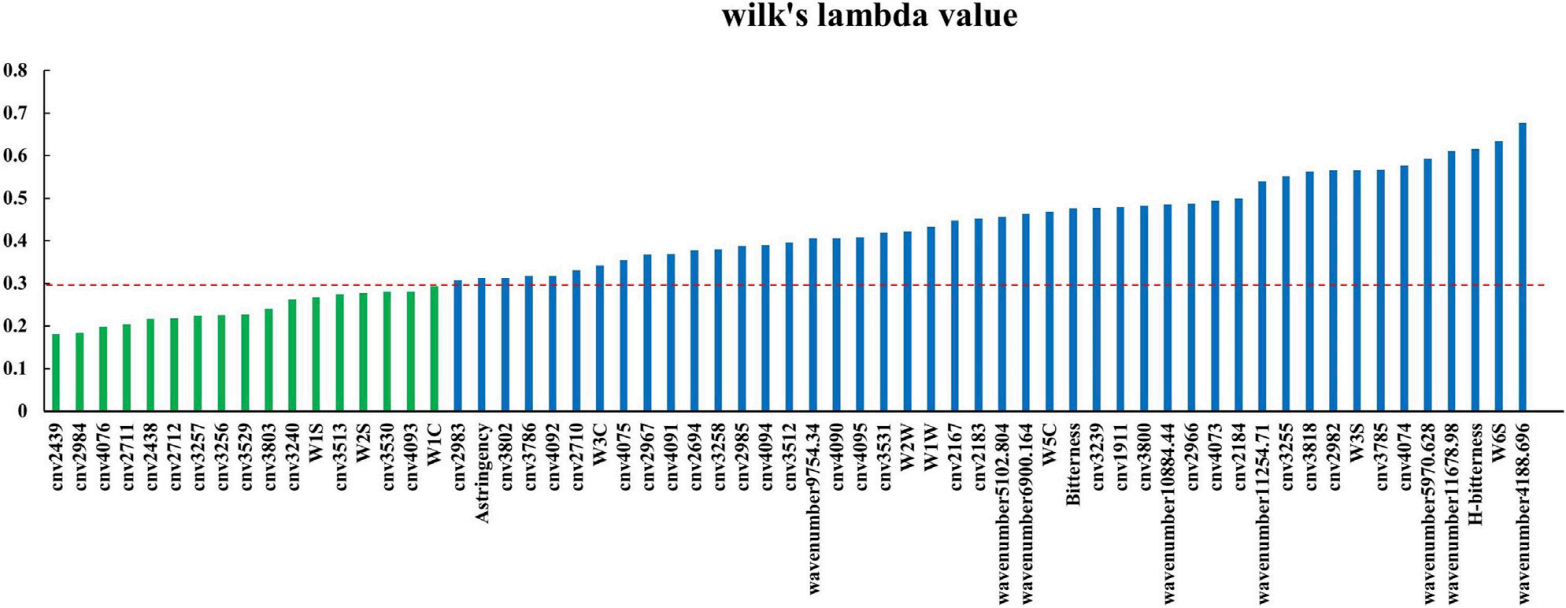
Wilk’s lambda value of original variables in the four-source data fusion PCA-DA model (cnv: color number value).

The variables that contributed the most to the classification of samples were selected if Wilk’s lambda value <0.3. We selected a total of 17 variables. The selected variables are shown in Figure 10, and the information they represent are listed in Table 5, which shows the contribution to the discrimination of authentic FC samples is significant.

**TABLE 5:** Variables selected based on Wilk’s lambda values and their response substances.

Variables	Response substances (information)
Color number value 2,439	L: 57.822, a: 4.244, b: 10.942
Color number value 2,984	L:69.632, a:2.25, b:18.817
Color number value 4,076	L: 92.679, a: 1.916, b: 17.697
Color number value 2,711	L:63.64, a:2.371, b:19.17
Color number value 2,438	L:57.535, a:2.516, b:19.565
Color number value 2,712	L:63.929, a:4.119, b:10.712
Color number value 3,257	L:75.524, a:2.147, b:18.497
Color number value 3,256	L:75.268, a:0.568, b:26.636
Color number value 3,529	L:81.065, a:0.459, b:26.233
Color number value 3,803	L:87.04, a:1.983, b:17.941
Color number value 3,240	L:71.156, a:8.649, b:21.178
W1S	alkane
Color number value 3,513	L:77.007, a:8.412, b:20.803
W2S	Alcohols, part of aromatic compounds
Color number value 3,530	L:81.324, a:2.059, b:18.207
Color number value 4,093	L:96.96, a: 3.919, b:15.452
W1C	aromatic hydrocarbons

By selecting variables based on the Wilk’s lambda value, we identified 14 among the 43 original variables of electronic eye data that contributed greatly to the classification of fusion data. Except for color number values 4,076, 2,711, 3,256, 3,529 and 3,803, the response values of the remaining 9 color number values were small. Among the 9 original variables of electronic nose data, 3 variables (W1S, W2S and W1C) showed a large contribution to the classification results in the data fusion model. Yet the W1S sensor had the largest response value, showing once again that the sensors that contribute the most to sample classification are not necessarily the ones with a high response value.

None of the electronic tongue sensors had a significant contribution to the classification results in fusion data.

We performed a *t*-test on the original data collected by the sensors with large contributions. On color number values 2,712 and 3,257 of the electronic eye, the four types of samples were significantly different (*p* < 0.05), while on the remaining 12 color number values there was no significant difference.

In the original data of four types of samples, FT and FU showed no significant difference on W1S and W2S, and FU and FD showed no significant difference on W1C. Yet, the model identification results were not based on a single sensor, but on the comprehensive results from multiple sensors.

## 4 Conclusion

We established a functional and effective method to determine the authenticity and identify the species of *Fritillariae cirrhosae* samples based on electronic nose, electronic tongue, and electronic eye sensors, NIR spectra, and mid-level data fusion technology. We proved the established PLS-DA model can accurately identify authentic and counterfeit FC samples. The identification model with the best performance was the four-source data fusion model (based on the fusion of electronic nose, electronic tongue, electronic eye data and NIR spectra). The positive identification rate of the model was as high as 98.75%.

In addition, the established PCA-DA model could effectively discriminate between species related to FC. The species identification model with optimal performance was based on electronic eye or on NIR spectra data or on four-source data fusion and its positive discriminant rate was 97.50% in three cases. The model explanation and analysis showed that the information collected by W1S, W2S and W1C sensors in the electronic nose, the Astringency sensor in electronic tongue, color number values 3,530 and 4,091 in the electronic eye and eight NIR characteristic wavelengths selected by CARS were the key quality information for the model to distinguish between the authentic and counterfeit FC samples and identify their species. Being able to extract such information from FC samples makes it possible to achieve a rapid evaluation of the quality of FC decoctions. We believe this study provides reference methods for the rapid evaluation of the quality of FC, as well as for the evaluation and control of the quality of other herbal samples.

## Data Availability

The original contributions presented in the study are included in the article/Supplementary Material, further inquiries can be directed to the corresponding authors.
