# Supplementary material for: Authenticity and species identification of Fritillariae cirrhosae: a data fusion method combining electronic nose, electronic tongue, electronic eye and near infrared spectroscopy
Source: Front Chem. 2023 Apr 28;11:1179039. doi: 10.3389/fchem.2023.1179039 (PMC10175593; doi:10.3389/fchem.2023.1179039)
Supplement: Supplementary file 1 [file Table1.DOCX]

TABLE 1 Results of human experience of the specialist identification

| **No.** | **Specialist**  **1** | **Specialist**  **2** | **Specialist**  **3** | **Specialist**  **4** | **Specialist**  **5** | **Specialist**  **6** | **Specialist**  **7** | **Specialist**  **8** | **No.** | **Specialist**  **1** | **Specialist**  **2** | **Specialist**  **3** | **Specialist**  **4** | **Specialist**  **5** | **Specialist**  **6** | **Specialist**  **7** | **Specialist**  **8** |
| --- | --- | --- | --- | --- | --- | --- | --- | --- | --- | --- | --- | --- | --- | --- | --- | --- | --- |
| 1 | 1 | 1 | 1 | 1 | 1 | 1 | 1 | 1 | 41 | 3 | 3 | 3 | 3 | 3 | 3 | 3 | 3 |
| 2 | 1 | 1 | 1 | 1 | 1 | 1 | 1 | 1 | 42 | 0 | 3 | 3 | 3 | 0 | 0 | 0 | 0 |
| 3 | 1 | 1 | 1 | 1 | 1 | 1 | 1 | 1 | 43 | 0 | 3 | 3 | 3 | 0 | 0 | 0 | 0 |
| 4 | 1 | 1 | 1 | 1 | 1 | 1 | 1 | 1 | 44 | 3 | 3 | 0 | 3 | 3 | 3 | 3 | 3 |
| 5 | 1 | 1 | 1 | 1 | 1 | 1 | 1 | 1 | 45 | 0 | 3 | 0 | 3 | 3 | 3 | 3 | 3 |
| 6 | 1 | 1 | 1 | 1 | 1 | 1 | 1 | 1 | 46 | 3 | 3 | 0 | 3 | 3 | 3 | 3 | 3 |
| 7 | 1 | 1 | 1 | 1 | 1 | 1 | 1 | 1 | 47 | 3 | 3 | 3 | 3 | 3 | 3 | 3 | 3 |
| 8 | 1 | 1 | 1 | 1 | 1 | 1 | 1 | 1 | 48 | 3 | 3 | 3 | 3 | 3 | 3 | 3 | 3 |
| 9 | 1 | 1 | 1 | 1 | 1 | 1 | 1 | 1 | 49 | 3 | 3 | 3 | 3 | 3 | 3 | 3 | 3 |
| 10 | 1 | 1 | 1 | 1 | 1 | 1 | 1 | 1 | 50 | 3 | 3 | 3 | 3 | 3 | 3 | 3 | 3 |
| 11 | 1 | 1 | 1 | 1 | 1 | 1 | 1 | 1 | 51 | 3 | 3 | 3 | 3 | 3 | 3 | 3 | 3 |
| 12 | 1 | 1 | 1 | 1 | 1 | 1 | 1 | 1 | 52 | 3 | 3 | 3 | 3 | 3 | 3 | 3 | 3 |
| 13 | 1 | 1 | 1 | 1 | 1 | 1 | 1 | 1 | 53 | 3 | 3 | 3 | 3 | 3 | 3 | 3 | 3 |
| 14 | 1 | 1 | 1 | 1 | 1 | 1 | 1 | 1 | 54 | 3 | 3 | 3 | 3 | 3 | 3 | 3 | 3 |
| 15 | 1 | 1 | 1 | 1 | 1 | 1 | 1 | 1 | 55 | 3 | 3 | 3 | 3 | 3 | 3 | 3 | 3 |
| 16 | 1 | 1 | 1 | 1 | 1 | 1 | 1 | 1 | 56 | 3 | 3 | 3 | 3 | 3 | 3 | 3 | 3 |
| 17 | 1 | 1 | 1 | 1 | 1 | 1 | 1 | 1 | 57 | 3 | 3 | 3 | 3 | 3 | 3 | 3 | 3 |
| 18 | 1 | 1 | 1 | 1 | 1 | 1 | 1 | 1 | 58 | 3 | 3 | 3 | 3 | 3 | 3 | 3 | 3 |
| 19 | 1 | 1 | 1 | 1 | 1 | 1 | 1 | 1 | 59 | 3 | 3 | 3 | 3 | 3 | 3 | 3 | 3 |
| 20 | 1 | 1 | 1 | 1 | 1 | 1 | 1 | 1 | 60 | 3 | 3 | 3 | 3 | 3 | 3 | 3 | 3 |
| 21 | 2 | 2 | 2 | 2 | 0 | 2 | 2 | 2 | 61 | 4 | 4 | 4 | 4 | 4 | 4 | 4 | 4 |
| 22 | 2 | 2 | 0 | 2 | 0 | 2 | 2 | 2 | 62 | 4 | 4 | 4 | 4 | 4 | 4 | 4 | 4 |
| 23 | 0 | 2 | 0 | 0 | 0 | 0 | 4 | 4 | 63 | 4 | 4 | 4 | 4 | 4 | 4 | 4 | 4 |
| 24 | 4 | 2 | 4 | 0 | 0 | 0 | 4 | 4 | 64 | 4 | 4 | 4 | 4 | 4 | 4 | 4 | 4 |
| 25 | 2 | 2 | 2 | 2 | 2 | 2 | 2 | 2 | 65 | 4 | 4 | 4 | 4 | 4 | 4 | 4 | 4 |
| 26 | 2 | 2 | 2 | 2 | 2 | 2 | 2 | 2 | 66 | 4 | 4 | 4 | 4 | 4 | 4 | 4 | 4 |
| 27 | 2 | 2 | 2 | 2 | 2 | 2 | 2 | 2 | 67 | 4 | 4 | 4 | 4 | 4 | 4 | 4 | 4 |
| 28 | 2 | 2 | 2 | 2 | 2 | 2 | 2 | 2 | 68 | 4 | 4 | 4 | 4 | 4 | 4 | 4 | 4 |
| 29 | 2 | 2 | 2 | 2 | 2 | 0 | 2 | 2 | 69 | 4 | 4 | 4 | 4 | 4 | 4 | 4 | 4 |
| 30 | 2 | 2 | 2 | 2 | 2 | 2 | 2 | 2 | 70 | 4 | 4 | 4 | 4 | 4 | 4 | 0 | 4 |
| 31 | 2 | 2 | 2 | 2 | 2 | 2 | 2 | 2 | 71 | 4 | 4 | 4 | 4 | 4 | 4 | 4 | 4 |
| 32 | 2 | 2 | 2 | 2 | 2 | 2 | 2 | 2 | 72 | 4 | 4 | 4 | 4 | 4 | 4 | 4 | 4 |
| 33 | 2 | 2 | 2 | 2 | 2 | 2 | 2 | 2 | 73 | 4 | 4 | 4 | 4 | 4 | 4 | 4 | 4 |
| 34 | 2 | 2 | 2 | 2 | 2 | 2 | 2 | 2 | 74 | 4 | 4 | 4 | 4 | 4 | 4 | 4 | 0 |
| 35 | 2 | 2 | 2 | 2 | 2 | 2 | 2 | 2 | 75 | 4 | 4 | 4 | 4 | 4 | 4 | 4 | 4 |
| 36 | 2 | 2 | 2 | 2 | 2 | 2 | 2 | 2 | 76 | 4 | 4 | 4 | 4 | 4 | 4 | 4 | 4 |
| 37 | 2 | 2 | 2 | 2 | 2 | 2 | 2 | 2 | 77 | 4 | 4 | 4 | 4 | 4 | 4 | 4 | 4 |
| 38 | 2 | 2 | 2 | 2 | 2 | 2 | 2 | 2 | 78 | 4 | 4 | 4 | 4 | 4 | 4 | 4 | 4 |
| 39 | 2 | 2 | 2 | 2 | 2 | 2 | 2 | 2 | 79 | 1 | 4 | 0 | 0 | 4 | 0 | 0 | 4 |
| 40 | 2 | 2 | 2 | 2 | 2 | 2 | 2 | 2 | 80 | 4 | 4 | 4 | 0 | 4 | 4 | 4 | 4 |

(“1” represents FT; “2” represents FU, “3” represents FD, “4” represents FUS and “0” represents unclassified.)
