# Supplementary figures and images for: Authenticity and species identification of Fritillariae cirrhosae: a data fusion method combining electronic nose, electronic tongue, electronic eye and near infrared spectroscopy
Source: Front Chem. 2023 Apr 28;11:1179039. doi: 10.3389/fchem.2023.1179039 (PMC10175593; doi:10.3389/fchem.2023.1179039)

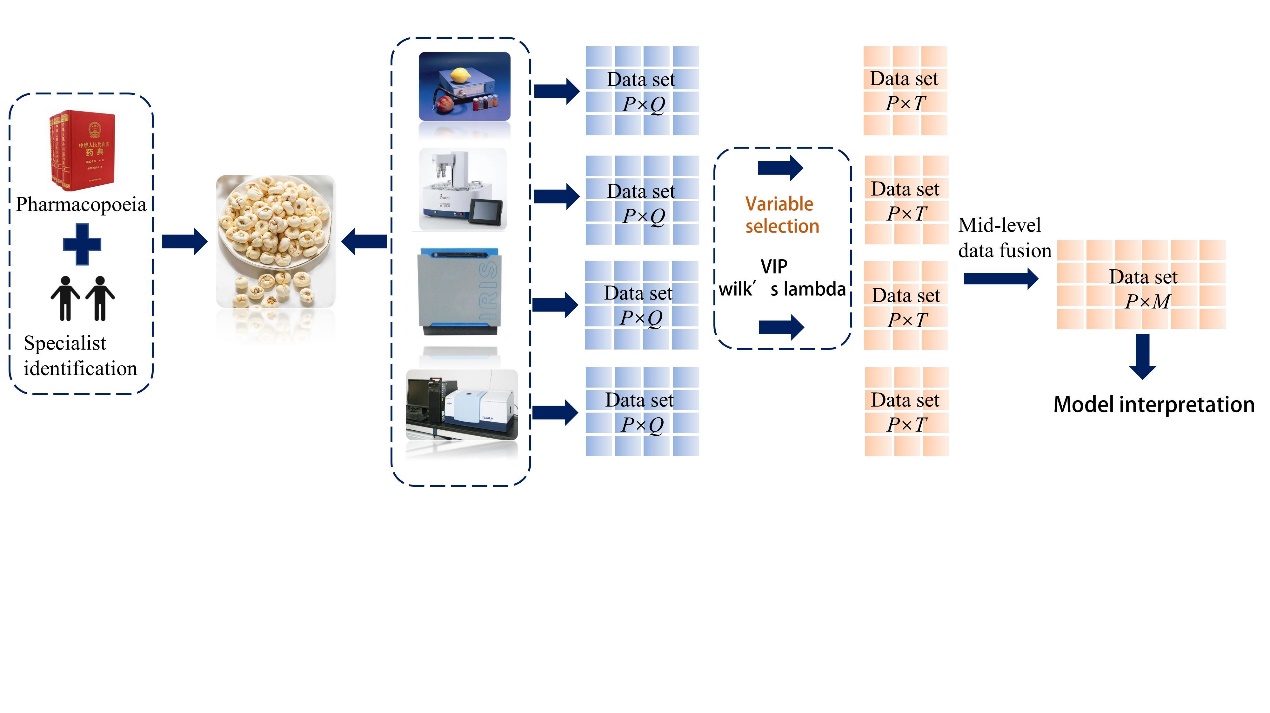

Supplement: Supplementary file 2 [file DataSheet1.DOCX]
